# Animal models of focal ischemic stroke: brain size matters

**DOI:** 10.3389/fstro.2023.1165231

**Published:** 2023-06-29

**Authors:** Blazej Nowak, Piotr Rogujski, Raphael Guzman, Piotr Walczak, Anna Andrzejewska, Miroslaw Janowski

**Affiliations:** ^1^Neurosurgery Department, John Paul II Western Hospital, Grodzisk Mazowiecki, Poland; ^2^Faculty of Medicine, Lazarski University, Warsaw, Poland; ^3^NeuroRepair Department, Mossakowski Medical Research Institute, Polish Academy of Sciences, Warsaw, Poland; ^4^Department of Neurosurgery, University of Basel, Basel, Switzerland; ^5^Program in Image Guided Neurointerventions, Center for Advanced Imaging Research, Department of Diagnostic Radiology and Nuclear Medicine, University of Maryland Marlene and Stewart Greenebaum Comprehensive Cancer Center, University of Maryland, Baltimore, MD, United States; ^6^Tumor Immunology and Immunotherapy Program, University of Maryland Marlene and Stewart Greenebaum Comprehensive Cancer Center, University of Maryland, Baltimore, MD, United States

**Keywords:** ischemic stroke, cerebral ischemia-reperfusion, small animal models, large animal models, middle cerebral artery occlusion, photothrombosis, endothelin-1, clinical translation

## Abstract

Stroke remains the second leading cause of death worldwide and the third cause of disability-adjusted life-years. Most strokes are ischemic in nature, meaning they are caused by the disruption of cerebral blood flow resulting from obstructed blood vessels. Reperfusion therapies such as thrombolysis with tissue plasminogen activator and endovascular mechanical thrombectomy are very effective and are becoming game changers for eligible patients. Despite these advances, the achieved effects are insufficient from the perspective of the entire population of stroke patients. Therefore, there is an urgent need to expand eligibility for reperfusion therapies and implement adjuvant therapeutic measures. Animal stroke models are at the forefront of these efforts, helping to untangle complex pathophysiology and providing valuable preclinical data to guide further clinical trials. Various stroke models are available, including direct blocking of cerebral arteries or using other means to recapitulate stroke pathophysiology. International advisory boards recommend initial *in vivo* experiments be performed in smaller animals, such as rodents. However, second testing would be more desirable in larger animals such as cats, pigs, dogs, and non-human primates. Due to larger cerebral volume, gyrencephalization, and higher white/gray matter ratio, large animals are crucial in translational stroke research. Animal stroke models differ in the time and complexity of the stroke induction procedure, the reproducibility rate, the level of similarity to the human condition, and the possibilities for analysis, imaging, and follow-up studies. The choice of the most appropriate stroke model may translate to better bench-to-bedside translation of preclinical stroke research; ideally, this choice should be based solely on scientific merit.

## 1. Introduction

Every year, October 29^th^ marks World Stroke Day (Sandercock, 2018). This initiative raises awareness about stroke, the second leading cause of death worldwide and the third cause of disability-adjusted life-years (Vos et al., 2020). Although the term stroke encompasses neurological injuries resulting from any vascular cause, 87% of human strokes are ischemic in nature (Benjamin et al., 2018; Mazuryk et al., 2021). Essentially, ischemic stroke is caused by the disruption of cerebral blood flow (CBF) resulting from the obstructed blood vessel. There are only two clinically approved therapies to restore blood flow in the occluded vessel: intravenous thrombolysis with tissue plasminogen activator (tPA) and endovascular mechanical thrombectomy (EMT). Developed initially using rabbits, tPA is a serine protease that dissolves blood clots (Matsuo et al., 1981). Unfortunately, both therapies can only be introduced within a narrow therapeutic window: (3-4.5 hours after the stroke event) for tPA (Bluhmki et al., 2009), which effectively applies to only 1-8% of stroke patients (Dhaliwal et al., 2019), and 6 hours for EMT, which can be further expanded till 24 hours for a narrow group of selected patients (Zivelonghi and Tamburin, 2018). Additionally, intracranial hemorrhage is a common complication following tPA treatment (Maïer et al., 2020). This limited set of therapeutic options has been the main driving force behind development of thousands of neuroprotective agents that have been tested in preclinical studies; however, none of them have been successfully translated into clinical practice. The failure of successful bench-to-bedside translation of new therapies for stroke energized the scientific community to establish the Stroke Therapy Academic Industry Roundtable (STAIR). This consortium issues guidelines regarding the quality and reproducibility of preclinical stroke research and, according to the 2009 update, recommends that initial *in vivo* experiments should be performed in rodents. More advanced preclinical testing studies are to be performed in gyrencephalic animals such as cats, pigs, and non-human primates (NHPs) (Fisher et al., 2009; Cai and Wang, 2016).

**Figure 1:**
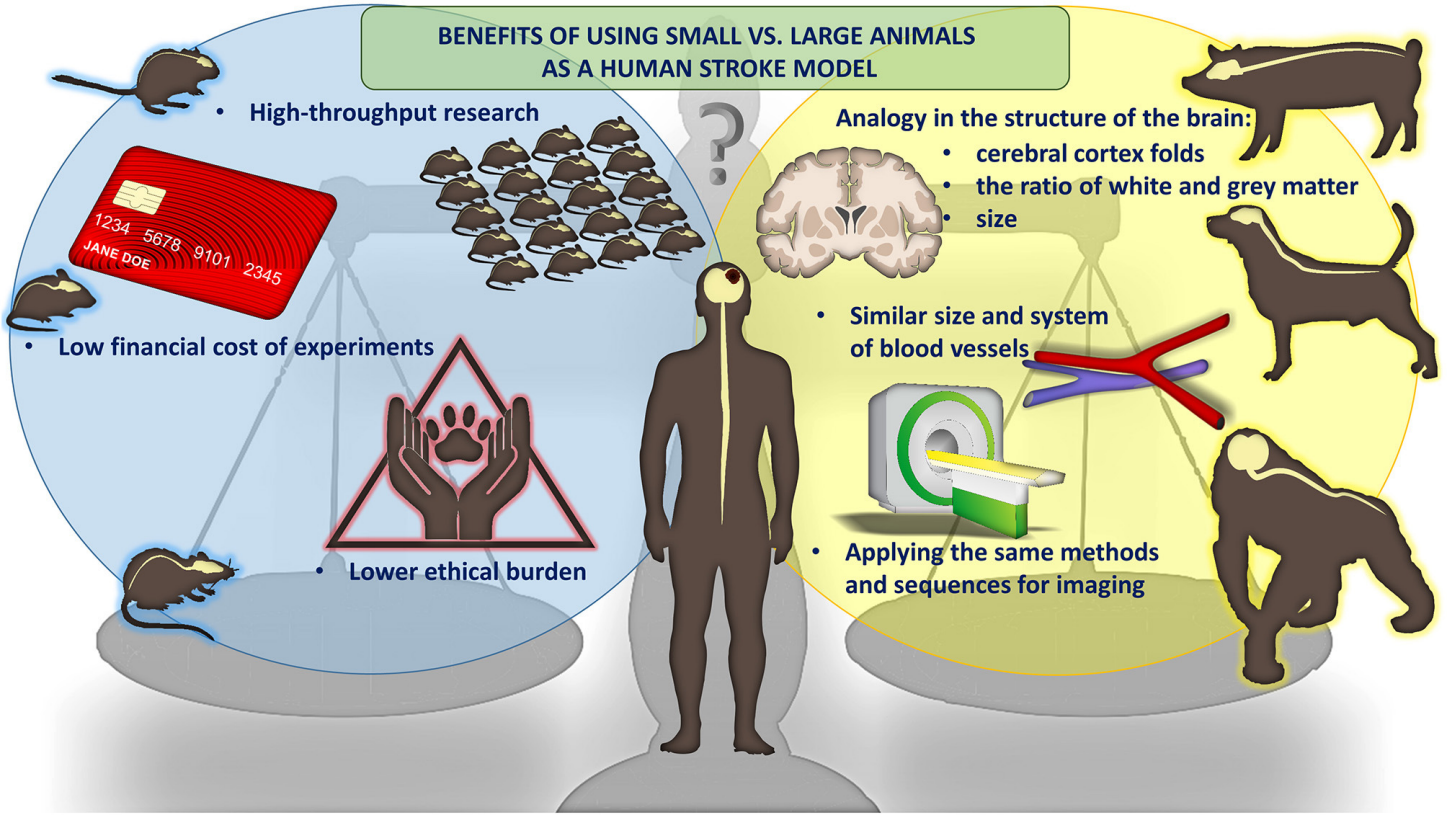
Size matters: advantages of small and large animals of stroke.

Since strokes are heterogenous diseases with complex etiologies, one of the most vital aspects of stroke studies is the selection of the most appropriate *in vivo* model. There are three main groups of stroke models, including the global, hemorrhagic, and focal models (Li and Zhang, 2021). Global models mimic the global cerebral ischemic event, which occurs when CBF is compromised and is usually triggered by a heart attack in adults or in neonatal hypoxic ischemic encephalopathy (HIE) in newborn. These models are generally easier to induce and are widely used to study the mechanisms of action of novel neuroprotectants (Woodruff et al., 2011). The hemorrhagic stroke models mimic the situation of spontaneous bleeding either into the brain parenchyma or subarachnoid space. It subsequently leads to the formation of hematoma and compression of brain structures (Zille et al., 2022). The induction of a focal ischemic stroke model, on the other hand, usually is based on a permanent or transient cerebrovascular occlusion, leading to a local disruption of cerebral blood flow (Kleinschnitz et al., 2015). Focal models seem to be the most clinically relevant so we will focus on them later in this article (Woodruff et al., 2011). An additional critical aspect we will discuss here is the phylogenetic complexity of the model species. Stroke can be modeled in small animals, allowing for greater throughput, lower cost and lower ethical burden. For larger, higher mammals, the advantage is much greater clinical relevance including more similar vasculature, brain size and white to gray matter ratio. Also, imaging is performed using the same methods and instruments as in human (Figure 1).

## 2. Transcranial occlusion

Transcranial models allow direct access to and closure of the superficial cerebral arteries at the base of the skull. Electrocoagulation of the artery causes permanent occlusion, whereas the use of removable microclips or sutures allows for transient occlusion and subsequent reperfusion.

In Robinson et al. (1975) was the first who described distal middle cerebral artery (MCA) occlusion *via* a craniectomy approach, which was developed in Sprague-Dawley rats. This was followed by Tamura et al. (1981), who used the same approach however, occluded the middle cerebral artery proximally, where it origins from the internal carotid artery (ICA). Modifying this technique includes ipsilateral ICA electrocoagulation to generate a tandem occlusion (Brint et al., 1988), or bilateral ICSs, the three-vessel occlusion, resulting in large hemispheric infarcts (Chen et al., 1986). Proximal M1 segment MCA occlusion produces reproducible cortical and basal ganglia infarcts; however, the more distally the artery is occluded, the smaller and more variable infarcts are due to collateral blood flow in the cerebral cortex.

Besides rodents, transcranial occlusion may also be used in large animals including dogs, sheep, pigs, and NHPs. In 1970, Hudgins described a method of direct MCA occlusion in squirrel monkeys *via* a transorbital approach with the total eyeball, optic nerve, and posterior orbital wall removal (Hudgins and Garcia, 1970). This method allows for direct proximal and distal M1 occlusion and anterior cerebral artery (A1) occlusion. However, the transorbital approach does not allow for distal MCA occlusion. Thus, the craniectomy model was developed to target distal M2 branches (Marshall and Ridley, 1996).

Craniectomy models are invasive, cause significant morbidity independent of stroke, and require microsurgical skills. On the other hand, it offers good reproducibility in infarct size, and neurological deficits, with relatively low mortality. However, neural and vascular structures may be directly damaged during the procedure; dura opening affects intracranial pressure and cerebrospinal fluid circulation, and may result in surgical site infection. In addition, the transorbital approach causes one-sided blindness, affecting the animals' neurobehavior assessment. The use of removable micro clips allows for reperfusion studies; however, it causes very rapid restoration of the blood flow rather than progressive reperfusion, which is observed in human stroke.

## 3. Endovascular filament middle cerebral artery occlusion

The goal of the endovascular filament Middle Cerebral Artery Occlusion (MCAO) is to block blood flow by a filament or suture placed within the artery lumen. Introduced by Koizumi et al. (1986), followed by many modifications of a kind, coating, length of monofilament, access route, rodent strains, and occlusion time (Longa et al., 1989), it became the most popular method in ischemic stroke studies, used in more than 40% of animal models (Howells et al., 2010). Technically, MCAO is relatively less invasive since it does not require craniectomy; however, first steps of the procedure require microsurgical exposure of the common, internal, and external carotid arteries. Next, through the ICA, a filament with a round tip is advanced into the MCA until it interrupts blood supply, which may be confirmed by laser doppler flowmetry. Moreover, besides MCA, anterior and posterior cerebral arteries are usually occluded. The model may be used for permanent or transient ischemia, depending on the time of filament placement within the artery lumen. Infarct area and severity depend on occlusion time. Short-lasting MCA occlusion induces disseminated neuronal injury in the striatum, most vulnerable to ischemic injury, because of lack of collateral flow (Garcia et al., 1995). More prolonged or permanent occlusions produce severe hemispheric infarcts with minimal penumbra territory, also affecting the hypothalamus, which causes hyperthermia in rats, a complication rarely seen in humans (Li et al., 1999). The significant drawback of intraluminal occlusion is the reperfusion process. In humans, vessel occlusion is frequently incomplete because of gradual, spontaneous reperfusion (Zanette et al., 1995). In contrast, after filament retrieval, the blood flow is rapidly restored to the baseline level. However, this mimics the clinical scenario with mechanical thrombectomy when a blood clot is removed using endovascular devices, and blood flow is rapidly restored.

Because of its lower invasiveness, intraluminal MCAO models were also adapted to large animals. In Bihel et al. (2010) showed the feasibility of inducing transient and permanent intraluminal MCAO in marmoset, which resulted in subcortical and cortical brain ischemia evidenced by magnetic resonance imaging (MRI), histology, immunohistochemistry, and long-lasting functional deficits. Especially, MRI monitoring showed a lesion evolution similar to humans rather than rodents. Extension of the intraluminal MCAO model in rodents are endovascular occlusions performed under digital subtraction angiography in large animals (see later).

## 4. Embolic occlusion

Embolic occlusion models fall into two major categories: thromboembolic and non-thromboembolic. Thromboembolic models are based on applying autologous or allogenic spontaneous blood clots or thrombin-induced blood clots. Blood is obtained from a tested animal (autogenous) or another species, usually human (allo-/xenogeneic), left for clot formation and intraarterially injected to cause vessel occlusion.

In Kudo et al. (1982) described thromboembolic occlusion in rats, generated by blood clot injection into the internal carotid artery (Kudo et al., 1982). The surgical technique with common, internal, and external carotid arteries exposition was the same as with the intraluminal filament model. Distribution of infarct was extensive and variable, from large hemispheric occlusions, within different territories of the ipsilateral hemisphere: parietotemporal cortex, hippocampus, basal ganglia, reaching even opposite hemisphere. To provide more accuracy and reproducibility, the intracarotid injection was replaced by direct MCA catheterization in rats (DiNapoli et al., 2006). By microsurgical exposition, a microcatheter was inserted into the carotid artery and introduced into MCA. Catheter position and artery occlusion were confirmed by a laser Doppler probe placed on the cranium at the temporal region. Ischemia was monitored for 120 min, tPA was administered IV, and a Doppler probe again confirmed MCA blood flow restoration.

Because most human ischemic strokes have a thromboembolic origin, this model is excellent for thrombolytic therapy studies. It is worth mentioning that the only approved thrombolytic drug, alteplase, was developed in rabbit embolic stroke model (Zivin, 1988). The response of the thromboembolic models to tPA depends on the composition and volume of the emboli and animal strain. Thrombin-induced clots are fibrin-rich and have a longer response to tPA than spontaneously forming clots. Also, different animals have different susceptibility to alteplase; *in vitro*, human tPA can dissolve over 95% human clots, 80% primate clots, and only 10% rat clots, consequently choosing rats for thrombolysis studies; alteplase dose should be tenfold higher (Korninger and Collen, 1981).

Embolic occlusion models may also be induced by artificial micro/macrospheres injections directly into CCA, ICA, MCA, in small and large animals. However, due to significant progress in interventional neuroradiology techniques and imaging, endovascular occlusion models in large animals are more precise and reproducible (see below). Microspheres induced-stroke results in multifocal and heterogenous infarcts in the parietotemporal cortex, corpus callosum, hippocampus, and basal ganglia (Miyake et al., 1993). Development of the lesion is gradual and slow, may last 24-48h, and mimics transient ischemic attack in humans. In contrast, embolization models are more reproducible and produce more focal ischemic infarcts similar to intraluminal suture occlusion, without the risk of developing hypothalamic injury and hyperthermia. The substantial difference between thromboembolic and artificial sphere models is that artificial spheres do not dissolve, causing permanent occlusion.

## 5. Endovascular occlusions in large animal models

Many methods of inducing vessel occlusion in small animals have been translated to large animals, such as thromboembolic and intraluminal suture-based techniques, and even further improved by the use of endovascular coils and balloons.

Besides gyrencephalization and favorable white/gray matter ratio, large animals have larger vascular diameters than rodents, making them suitable candidates for endovascular models (Taha et al., 2022). Intracranial vessel accessibility also depends on head-neck vascular anatomic configurations. Rete mirabile (RM) is a complex of anastomosing vessels located proximal to the intracranial ICA in pigs and sheep. Humans, NHPs, and dogs do not have RM, and their extracranial-intracranial vascular anatomy allows for precise microcatheter manipulations, producing accurate and reproducible ischemic infarcts. An essential advantage of endovascular techniques is the possibility of delivering targeted drugs, or stem cells, during mechanical thrombectomy procedures in the same setting as well as in sub-acute or chronic stroke phases.

**Figure 2:**
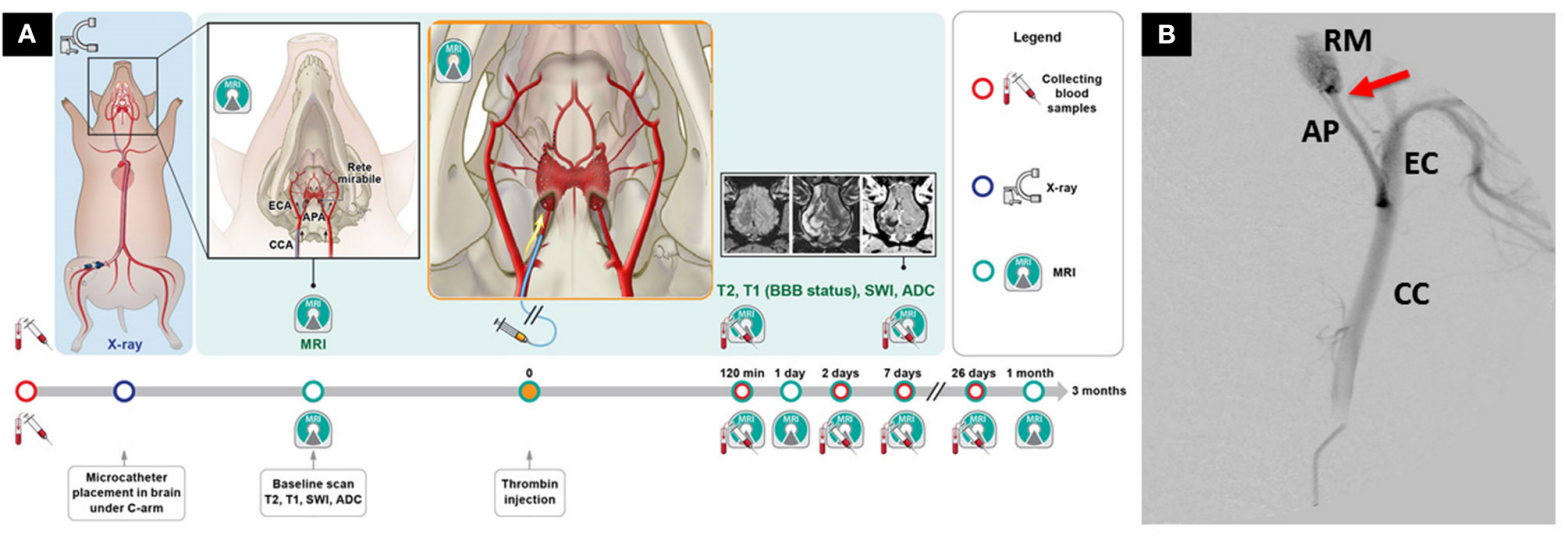
Novel endovascular model of focal ischemic stroke in a large animal. **(A)** Intra-arterial administration of thrombin mixed with gadolinium allows real-time visualization of the occlusion with magnetic resonance imaging (MRI). **(B)** Placement of the microcatheter proximally to *rete mirabile* allows trans-catheter perfusion of the ipsilateral hemisphere, as visualized by contrast-enhanced perfusion MRI. The study has overcome the long-lasting challenge of inducing endovascular stroke model in pigs. Reproduced from Golubczyk et al., 2020, licensed under CC BY 4.0. APA/AP, ascending pharyngeal artery; BBB, blood-brain-barrier; CCA/CC, common carotid artery; ECA/EC, external carotid artery; RM, *rete mirabile*. Red arrow indicates placement of the microcatheter.

Historically, due to RM, endovascular thromboembolic models were not available in swine. However, in 2020 Golubczyk et al. (2020) introduced an endovascular thrombin-induced ischemic stroke model in swine. Under digital subtraction angiography a catheter was introduced to ascending pharyngeal artery just proximal to RM. This pioneering experiment was conducted under the guidance of interventional magnetic resonance imaging. First, gadolinium contrast was pre-injected via an arterial catheter to assess perfusion areas of the brain in the ipsilateral hemisphere. Next, a thrombin-gadolinium mixture was injected, and dynamic GE-EPI MRI scans showed hyperintense regions in the ipsilateral hemisphere indicating vascular occlusion. This model was later used to study the therapeutic potential of neuroprotective agent glycolic acid with combined systemic and intra-arterial administration in the acute phase of stroke (Chovsepian et al., 2022). This experiment demonstrated the possibility of inducing endovascular ischemic stroke in swine (Figure 2), which raises less ethical controversies than NHPs and dogs.

Endovascular occlusion models provide precise and reproducible methods to induce focal, permanent, or transient ischemia in any desired brain territory. However, these techniques require costly infrastructure including an X-ray angiography suite for catheterization, microcatheters, and microwires. These are significant limitations because of economic and logistical factors, further compounded by the ethical issues.

## 6. Photothrombosis model

The photothrombosis model of ischemic stroke is induced by localized intravascular photo-oxidation. The procedure relies on intraperitoneal (in mice) (Lee et al., 2007) or intravenous (in rats and larger animals) (Kuroiwa et al., 2009; Khateeb et al., 2022) administration of a photosensitive dye, such as rose Bengal or erythrosine B, followed by focal irradiation of the cranium. It generates free oxygen radicals, which lead to endothelial damage, platelet aggregation, and blood clot formation (Braeuninger and Kleinschnitz, 2009; Li and Zhang, 2021). The major advantages of the photothrombosis model are minimal invasiveness and relatively simple methodology, high reproducibility of the ischemic lesion, and low mortality. Also, as recently shown by Weber et al., photothrombosis can be effectively applied to study blood-brain barrier leakage even beyond the acute stroke stage (Weber et al., 2020). However, due to the lack of ischemic penumbra, this model does not ideally reflect the clinical scenario, where the formation of cytotoxic edema is a hallmark of focal ischemic stroke and a common therapeutic target. Besides the lack or marginal local collateral blood flow (no penumbra), reperfusion is also not observed after photothrombosis (Uzdensky, 2018). However, some improvements were achieved by UV laser-based clot dissolution (Watson et al., 2002) and modulation of neutrophil extracellular trap formation (NETs). NETs are extracellular DNA strings secreted by neutrophils related to platelet aggregation and thrombi formation, hence contributing to tPA treatment resistance (Peña-Martínez et al., 2019).

Photothrombosis was initially developed in rats to study cortical ischemia (Watson et al., 1985) and was soon adapted to other small animals such as mice and Mongolian gerbils (Kuroiwa et al., 2016). The benefit of inducing photothrombosis in smaller animals is minimal invasiveness (no need for craniotomy); however, additional live brain imaging with modalities such as intravital microscopy would still necessitate bone removal (Nowak et al., 2022). The protocol for smaller animals was significantly improved with a ring-shaped laser beam, which upon dye photoactivation, led to the formation of a penumbra-mimicking ischemic zone (Wester et al., 1995). Further, photothrombosis was made possible beyond the cortex in the subcortical structures such as the internal capsule (Kim et al., 2014; Han et al., 2017) or striatum (Lv et al., 2018). Recently, Fukuda et al. (2022) established a photothrombosis protocol for mice that can specifically target even a single vessel in the cortical parenchyma by two-photon excitation with a success rate of 84.9 ± 1.7% and an irradiation time of < 80 s (Fukuda et al., 2022).

Due to the complexity of the brain structure and limited light penetration, adaptation of the photothrombosis model to larger animals usually requires craniotomy before illumination. It makes the procedure more invasive and limits both throughput and reproducibility. Nonetheless, photothrombosis was already successfully introduced in larger animals such as piglets (Kuluz et al., 2007) and NHPs, including cynomolgus macaques (Maeda et al., 2005), marmosets (Ikeda et al., 2013), and rhesus monkeys (Zhang et al., 2021). Notably, Yang et al. (2020) used rhesus monkeys after photothrombotic stroke to demonstrate that circular RNA SCMH1 promotes functional recovery by enhancing neuronal plasticity, inhibiting glial activation and peripheral immune cell infiltration (Yang et al., 2020). Recently, a piglet-based photothrombotic stroke model was used to test a hand-held transcranial photoacoustic imaging device. Authors concluded that oxygen saturation metric alone could be used to identify regional lesions within 2 h after stroke induction in a gyrencephalic brain (Kang et al., 2021).

## 7. Endothelin-1 model

The endothelin-1 model of focal ischemic stroke is induced by applying a strong, reversible vasoconstrictor endothelin-1. This 21-amino acid peptide causes significant CBF reduction, leading to local ischemia with negligible edema followed by gradual reperfusion. The strength and duration of endothelin-1-induced vasoconstriction are strictly dose-dependent (Traverse et al., 1996) and can be reversed, hence progressing to the reperfusion phase (Macrae et al., 1993). Endothelin-1 can be administered: topically onto the brain surface, through intracerebral injection, or directly into the exposed artery. Topical application induces a semicircular infarction involving all cortical layers (Fuxe et al., 1997); however, such delivery route causes uncontrollable diffusion, being a source of variability (Trotman-Lucas and Gibson, 2021). Meanwhile, direct infusion into the MCA or stereotaxic injection into the adjacent cortex endothelin-1 causes a rapid reduction of CBF in various brain areas, including the corpus callosum and caudate nucleus (Sharkey, 1993).

The endothelin-1 model was first established in rats (Li and Zhang, 2021) and was the first stroke model inducible in conscious animals (Sharkey, 1993), with cerebral infarctions comparable to those achieved in anesthetized MCAO models (Abeysinghe and Roulston, 2018). Interestingly, endothelin-1 is around four times more effective in freely moving than in anesthetized rats (Bogaert et al., 2000). The endothelin-1 model has prominent advantages, including relatively low invasiveness; flexible selection of infarct area; reversibility, good spatial control, and low animal mortality. Unfortunately, endothelin-1 is much less potent in mice where it produces insignificant infarctions (Horie et al., 2008), likely due to difference in endothelin-1 receptor expression pattern in mice (Wang et al., 2020), which effectively limits mice-based studies. Meanwhile, larger animals demonstrate excellent utility for endothelin-1-based stroke induction, as shown in pigs (Zhang et al., 2016), and NHPs, including marmosets (Virley et al., 2004) and rhesus monkeys (Dai et al., 2017). Recently, Boghdadi et al. utilized the endothelin-1-induced marmoset stroke model to transcriptomically characterize astrocytes in the infarcted visual cortex, demonstrating NogoA-mediated anti-inflammatory response limiting macrophage infiltration into the surviving parenchyma (Boghdadi et al., 2021). However, despite these promising results, aside from its vasoconstrictive properties, endothelin-1 is also involved in neural transmission and modulation, and endothelin-1 receptors are also expressed by neurons and astrocytes (Giaid et al., 1989; Nakagomi et al., 2000). Therefore, endothelin-1 administration can cause additional side effects which could interfere with the experiment outcome, affecting and complicating interpretation of the stroke study.

## 8. Ouabain model

**Figure 3:**
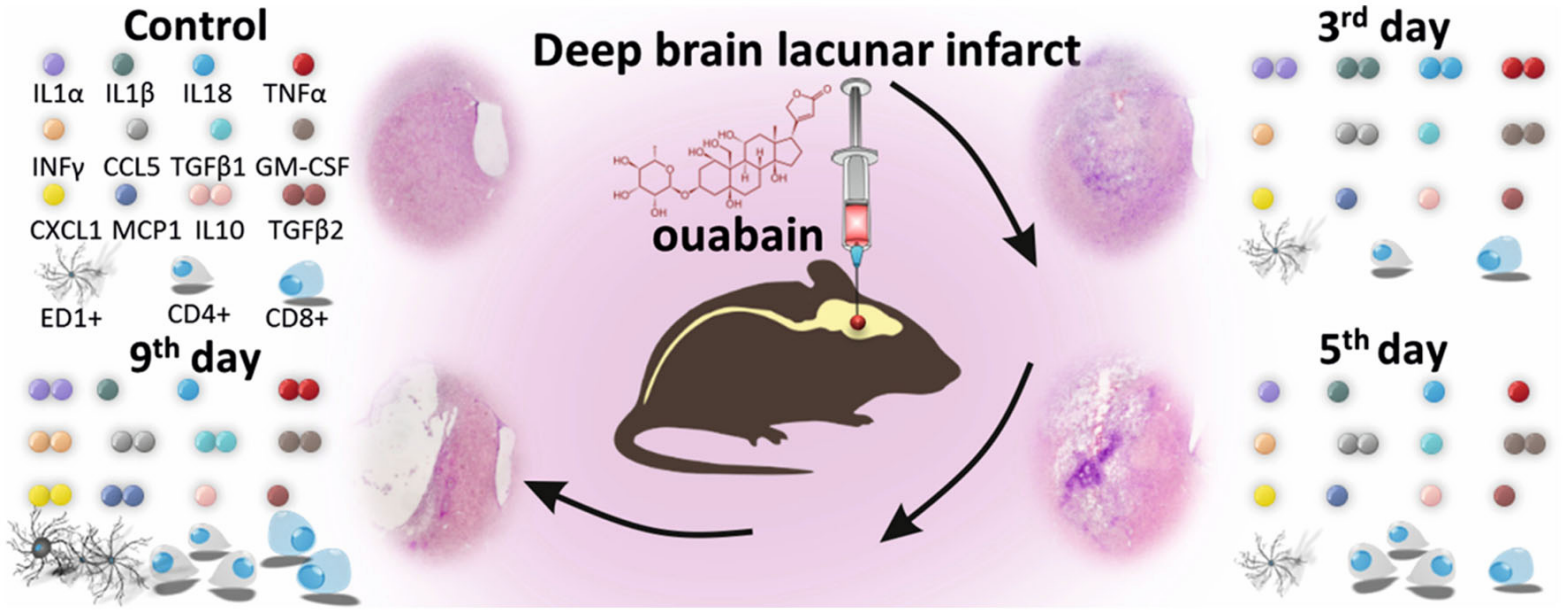
Ouabain-induced deep brain lacunar infarct model in a small animal. Intra-cerebral administration of ouabain, an inhibitor of Na/K-ATPase, causes cellular energy deprivation, inducing a stroke-like cascade of events, including severe immune response. Significant changes are visible in the histological picture of brain hemisphere and the level of pro-inflammatory and anti-inflammatory cytokines and chemokines, as well as composition of immune cell populations in the injured brain. Reproduced from Dabrowska et al., 2021, licensed under CC BY-NC-ND 4.0. The number of colored dots represents the level of each factor 3, 5, and 9 days after ouabain-induced brain injury, relative to control. The significant changes in the number of immune cells are reflected by multiplication of their graphical counterparts.

Alongside the many available, already described techniques of inducing focal stroke, one final that is worth mentioning is based on the administration of ouabain. Ouabain is an inhibitor of Na/K-ATPase, which causes cellular energy deprivation upon intracerebral delivery, mimicking a stroke-like cascade of events (Veldhuis et al., 2003; Janowski et al., 2008), including severe neuroinflammation (Dabrowska et al., 2021) (Figure 3). Moreover, as we have already shown, this model can be effectively implemented to study the therapeutic potential of intra-arterially delivered, modified mesenchymal stem cells (MSCs) (Andrzejewska et al., 2020; Nowak et al., 2021) as well as MSC-derived extracellular vesicles (Dabrowska et al., 2019).

## 9. Discussion

An *in vivo* research model is established when a non-human animal species is used to mimic a human condition, usually after a standardized, external intervention. Each model has its strengths and weaknesses, affecting the induction time and complexity; reproducibility rate; level of similarity to the human condition; and potential for analytics, imaging, and follow-up studies. In a clinical scenario, ischemic stroke is usually caused by an acute blood vessel obstruction, likely affected by the patient's age, sex, overall condition, comorbidities, and all these features are challenging to reproduce in animal model. The transient vessel occlusion seems to mimic clinical situation more closely, additionally allowing to precisely control the occlusion period hence reperfusion, which could be valuable for therapeutic window screening, and especially if induced in diabetic or hypertensive animal. Meanwhile, model animals are usually grouped by size and brain anatomy. They include smaller animals with lissencephalic brains, such as mice, rats, or gerbils, and larger animals with more human-like, gyrencephalic brains, including pigs, cats, and NHPs. Although smaller animals are usually easier to operate, manage and have lower cost, they demonstrate substantial anatomical differences from humans and have limited potential for behavioral evaluation. On the other hand, larger animals have white matter-rich brain content with strong tentorium cerebelli, more pronounced cerebral vasculature with collateral blood flow, and, especially in NHPs, can be subjected to reliable neurological assessment; however, the main disadvantages are high costs and ethical issues. Besides behavioral and neurological examination, appropriate imaging is obligatory to fully evaluate the study subject f.ex., regeneration potential of transplanted stem cells. Larger animals can often be analyzed with human-scale equipment such as computed tomography, magnetic resonance or positron emission tomography; however, it does not allow for detailed evaluation of cell viability and function. This drawback can be overcome by imaging techniques used in small animals such as bioluminescence, intravital microscopy and magnetic resonance microscopy (Driehuys et al., 2008; Nowak et al., 2022). Photon emission by luciferase-expressing cells is a principle of bioluminescence, which allows detection of living cells. Because of photon absorption in soft tissue and bones, it is only used in small animals. Intravital microscopy shows cells' function at cellular and subcellular level in living animal through small skull window. Hardware and software development expanded MRI to microscopic scale, allowing observation of brain structures 10–20 micrometers in size in magnetic resonance microscopy. The choice of animal model and imaging technique depends on the study type and should ideally be based solely on scientific merit. However, other non-scientific criteria that must be considered are ethical issues and overall research costs, including equipment, animal care, and maintenance.

Regardless of stroke induction technique and animal choice, *in vivo* models are still at the forefront of the preclinical battle against stroke. Alarmingly, however, the devastating socio-economic impact of stroke on societies and individual households seems not to bother European legislators. Instead, with initiatives such as 2021/2784(RSP) (“*Resolution on plans and actions to accelerate the transition to innovation without the use of animals in research, regulatory testing and education*”), the European Parliament hinders further scientific advancements and future clinical trials in Europe. We urge the research community to speak up and educate European societies that such actions will only benefit non-European governments, research institutions, and companies, limit scientific independence, and ultimately bury the enormous potential of animal studies to advance science and benefit humanity.

## Author contributions

All authors listed have made a substantial, direct, and intellectual contribution to the work and approved it for publication.
